# AI赋能的专病管理平台在肺癌患者术后康复中的应用研究

**DOI:** 10.3779/j.issn.1009-3419.2025.102.11

**Published:** 2025-03-20

**Authors:** Mei LI, Hongbing ZHANG, Chunqiu XIA, Yuqi ZHANG, Huihui JI, Yi SHI, Liran DUAN, Lingyu GUO, Jinghao LIU, Xin LI, Ming DONG, Jun CHEN

**Affiliations:** ^1^300052 天津，天津医科大学总医院肺部肿瘤外科（李梅，张洪兵，夏春秋，张雨琪，纪慧慧，石怡，段丽然，郭玲玉，刘京豪，李昕，董明，陈军）; ^1^Department of Lung Cancer Surgery; ^2^天津市肺癌转移与肿瘤微环境重点实验室，天津市肺癌研究所（陈军）; ^2^Tianjin Key Laboratory of Lung Cancer Metastasis and Tumor Microenvironment, Tianjin Lung Cancer Institute, Tianjin Medical University General Hospital, Tianjin 300052, China

**Keywords:** 肺肿瘤, 术后管理, 人工智能, 专病管理, 咳嗽, 生活质量, Lung neoplasms, Postoperative management, Artificial intelligence, Disease-specific management, Cough, Quality of life

## Abstract

**背景与目的:**

肺癌是我国发病率和死亡率最高的恶性肿瘤。随着国民健康意识的提升和低剂量螺旋计算机断层扫描（computed tomography, CT）的普及，肺癌早期诊断率逐年提高。胸腔镜微创手术凭借创伤小、恢复快等优势已成为首选术式，然而患者出院后的功能恢复仍需重点关注。传统随访模式存在标准化程度低、医疗资源消耗大、患者负担重等问题。基于人工智能（artificial intelligence, AI）的专病管理平台为患者出院后随访开辟了新途径。本研究通过对463例肺癌术后患者实施AI平台随访，深入分析常见问题及其解决方案，旨在对潜在的并发症进行早期干预、提升患者生活质量，同时推进AI技术在医疗领域的应用。

**方法:**

利用AI平台整合科普视频、医护团队与AI助手协作管理，记录健康日志、评估健康状况、实施个性化干预，并通过Leicester咳嗽问卷监测患者术后康复状况。采用独立变量t检验及单因素方差分析探究肺癌术后咳嗽的原因。

**结果:**

患者出院后7 d问题发生率最高，出院后14 d症状明显缓解。性别、吸烟史和手术方式是影响术后咳嗽恢复的关键因素。女性出院后7 d内咳嗽多于男性（P<0.01），而老年患者出院后14 d内咳嗽发生率低于年轻患者（P=0.03）。通过AI平台实施阶段性干预，患者的咳嗽、疼痛和睡眠问题得到了显著改善。

**结论:**

AI专病管理平台在提升肺癌术后管理效率和患者自我管理能力方面展现出较好的应用价值，尤其在分阶段管理术后咳嗽方面取得显著成效。未来结合可穿戴设备的应用，有望实现更精细化、个体化的术后康复管理，并推动AI技术在多学科医疗领域的广泛应用。

## Abstract (en)

**Background and objective:**

Lung cancer is the leading malignancy in China in terms of both incidence and mortality. With increased health awareness and the widespread use of low-dose computed tomography (CT), early diagnosis rates have been steadily improving. Surgical intervention remains the primary treatment option for early-stage lung cancer, and video-assisted thoracoscopic surgery (VATS) has become a common approach due to its minimal invasiveness and rapid recovery. However, post-discharge recovery remains incomplete, underscoring the importance of postoperative care. Traditional follow-up methods, lack standardization, consume significant medical resources, and increase the burden of the patients. Artificial intelligence (AI)-driven disease management platforms offer a novel solution to optimize postoperative follow-up. This study followed 463 lung cancer surgery patients using an AI-based platform, aiming to identify common postoperative issues, propose solutions, improve quality of life, reduce recurrence-related costs, and promote AI integration in healthcare.

**Methods:**

Using the AI disease management platform, this study integrated educational videos, collaboration between healthcare teams and AI assistants, daily health logs, health assessment forms, and personalized interventions to monitor postoperative recovery. The postoperative rehabilitation status of the patients was assessed by the Leicester Cough Questionnaire (LCQ-MC). Two independent t-test and one-way ANOVA were used to analyze the causes of postoperative cough in lung cancer.

**Results:**

Most issues occurred within 7 d post-discharge, significantly declined on 14 d post-discharge. Factors such as gender, smoking history, and surgical approaches were found to influence cough recovery. The incidence of cough on 7 d post-discharge in females was higher than that in males (P<0.01), while the incidence of cough on 14 d post-discharge in elderly patients was lower than that in young patients (P=0.03). The AI-based platform effectively addressed cough, pain, and sleep disturbances through phased interventions.

**Conclusion:**

The AI-based platform significantly enhanced postoperative management efficiency and the self-care capabilities of the patients, particularly in phased cough management. Future integration with wearable devices could enable more precise and personalized postoperative care, further advancing the application of AI technology across multidisciplinary healthcare domains.

据2022年国家癌症中心统计，我国肺癌年新发病例达82.81万例，死亡65.7万例，均居恶性肿瘤首位^[1]^。近年来，随着国民健康意识的提升和低剂量螺旋计算机断层扫描（computed tomography, CT）的普及，肺癌早诊率逐年提高。外科手术是早期肺癌的首选治疗方式^[2]^，其中胸腔镜微创手术凭借创伤小、恢复快、并发症少等优势，为患者快速康复提供了保障。目前患者住院时间明显缩短，多数患者出院时功能尚未完全恢复，因此出院后管理成为临床工作的重点。当前，国内外针对肺癌术后患者的随访方式主要包括电话、微信及门诊随访等，但传统医护主导的出院后管理存在不足，患者遇到问题常需返院就诊或通过互联网咨询，存在医护管理不连续、医疗资源耗费大、患者负担重等问题。因此，如何有效开展肺癌患者的出院后管理成为亟待解决的临床难题。

在大数据时代，人工智能（artificial intelligence, AI）技术正深刻变革医疗领域。将AI技术引入出院后管理，不仅能提升患者的自我照护能力，也为临床工作提供了便利。鉴于目前肺切除术后缺乏标准化随访方案，亟需建立规范化的随访流程^[3,4]^，AI驱动的专病管理服务平台应运而生。该平台整合了AI、互联网、大数据及可穿戴设备等先进技术，为公立医院提供全方位的专病慢病信息平台建设、医疗辅助及运营管理服务。平台通过健康科普推送、在线咨询、医护直播、线下就医等多元化服务模式，实现对患者健康状况的实时监测和个性化干预，打造全病程、精细化的标准化管理闭环，促进医疗资源高效利用和护理路径标准化发展。

本研究通过AI专病管理平台对463例肺癌术后患者进行出院后随访管理，对肺癌患者的术后康复过程进行了系统性总结，并归纳了患者术后康复常见的问题。

## 1 资料与方法

### 1.1 研究人群

本研究收集了2024年4月1日至7月31日在天津医科大学总医院肺部肿瘤外科接受手术治疗的患者。纳入标准：（1）接受肺部手术治疗；（2）病理证实为肺癌。排除标准：（1）年龄<18周岁；（2）不能或不愿接受AI专病管理平台随访。最终有463例患者纳入研究。

### 1.2 研究方法

#### 1.2.1 AI专病管家服务内容

AI专病管家以治疗组为单位，每组由主管医生、护理人员和AI助手组成，以对话形式为患者提供服务。AI助手通过每日健康日志分析，自动推送注意事项和教学视频，并在必要时提醒医护团队介入，实时提供精准、高效的服务，同时支持智能数据分析与决策优化（图1）。教学视频包括20个普适视频（如伤口消毒方法、术后饮食原则等）和7个针对性视频（如皮下气肿、痰中带血等）。同时，设有专业医护团队直播线上答疑。

**图1:**
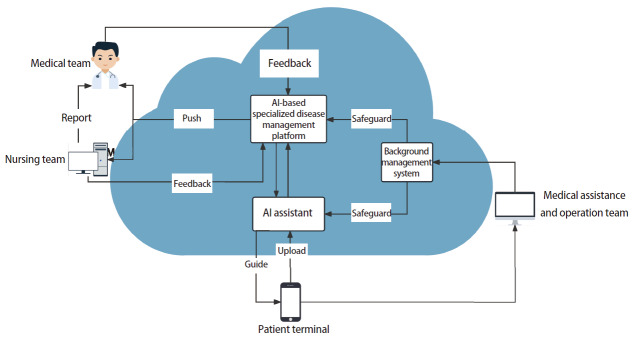
AI专病服务平台架构。患者将问题上传后，AI助手针对问题进行初步解答，并上传至云平台。通过AI算法，平台选择性推送问题至医师或护理团队，得到反馈后推送相应内容至患者。运行过程中涉及患者权益、信息运行或故障等问题通过医疗辅助与运营团队反馈解决。

#### 1.2.2 基线问卷设计

内容包括患者一般资料：身高、体重、吸烟史、既往肺疾病史等，在专病管理中对应患者填写健康评定并作为基础信息生成患者健康评估报告。

#### 1.2.3 健康日记及周期患者健康评定内容方案

针对手术后康复期的主要问题，出院后7、14 d，通过AI问答形式，使用AI系统患者自行填写评定量表，涉及疼痛、咳嗽、咳痰、疲乏气短、睡眠、饮食，Leicester咳嗽问卷出具健康报告，由医护人员审核后，给出相应治疗策略。康复期数据通过出院后7、14 d填写系统推送的评定量表收集，覆盖疼痛、咳嗽、咳痰、疲乏气短、睡眠、饮食等方面，并据此生成健康报告。Leicester咳嗽问卷涵盖躯体、心理、社会三方面，共19条目，采用Likert 7级评分，总分3-21分，分数越高，咳嗽影响越小，生活质量越高，该量表具有良好的信效度，Cronbach alpha系数为0.942。

#### 1.2.4 随访时间与观察指标

随访周期为出院后0-14 d。观察指标包括术后常见症状（咳嗽、咳痰、疲乏气短、疼痛）、并发症（气胸、皮下气肿、肺感染、胸腔积液、伤口愈合不良）以及饮食、睡眠等问题的发生时间、持续时间、干预效果和患者满意度等。被研究者的末次随访时间为出院后14 d，研究截止时间为2024年12月31日。

### 1.3 统计学方法

本研究使用SPSS 24.0软件进行数据统计分析。计量资料用均数±标准差（Mean±SD）表示，计数资料用率（%）表示，使用两独立变量t检验和单因素方差分析进行组间差异比较。使用双侧检验，P<0.05为差异具有统计学意义。

## 2 结果

### 2.1 肺癌术后患者出院后生活质量现状

2024年4月1日至7月31日共有463例患者参与AI专病管家随访，其中男性182例（39.31%），女性281例（60.69%）；65岁以上164例（35.42%），身体质量指数（body mass index, BMI）>23.9 kg/m^2^者223例（48.16%）；341例无吸烟史（73.65%），369例无肺癌家族史（79.70%），232例无合并症（50.11%）。手术方式以肺楔形切除为主（267例，57.67%），343例（74.08%）为单个肺结节切除，372例（80.35%）有2个胸部切口，具体见表1。肺切除术后对患者心理状态、生理状态、咳嗽等进行分析。患者在出院后7与14 d间的心理状态未见明显改善（P=0.42，表2），表明咳嗽问题在较长时间跨度上对患者造成了困扰；躯体难度方面有改善（P=0.03）。

**表1:** 肺切除术后患者一般人口学资料（n=463）

Characteristics		n (Percentage)
Gender	Male	182 (39.31%)
	Female	281 (60.69%)
Age	≤65 yr	299 (64.58%)
	>65 yr	164 (35.42%)
BMI	<18.5 kg/m^2^	14 (3.03%)
	18.5-23.9 kg/m^2^	226 (48.81%)
	>23.9 kg/m^2^	223 (48.16%)
Length of stay	≤3 d	190 (41.03%)
	4-6 d	247 (53.35%)
	≥7 d	26 (5.62%)
Smoking history	No	341 (73.65%)
	Yes	122 (26.35%)
Family history of lung tumors	No	369 (79.70%)
	Yes	94 (20.30%)
Complication	No	232 (50.11%)
	Yes	231 (49.89%)
Surgical approach	Lobectomy	142 (30.67%)
	Segmentectomy	46 (9.94%)
	Wedge resection	267 (57.67%)
	Septum resection	8 (1.72%)
Number of nodules removed	0	8 (1.73%)
	1	343 (74.08%)
	≥2	112 (24.19%)
Pathological results	Benign tumor	63 (13.60%)
	Malignant tumor	392 (84.67%)
	Others	8 (1.73%)
Lobe involved	1	377 (81.42%)
	≥2	78 (16.85%)
	Others	8 (1.73%)
Surgical side	Left side	176 (38.01%)
	Right side	274 (59.18%)
	Both sides	5 (1.08%)
	Others	8 (1.73%)
Number of incisions	Single hole	79 (17.06%)
	Two holes	372 (80.35%)
	Thoracotomy	4 (0.86%)
	Others	8 (1.73%)

BMI: body mass index.

**表2:** Leicester咳嗽问卷得分分析

Index	7 d post-discharge (n=275)	14 d post-discharge (n=218)	P
Total	15.54±3.15	16.31±2.97	0.09
Physiological dimension	5.18±1.11	5.50±1.07	0.03
Psychological dimension	5.12±1.22	5.31±1.14	0.42
Social dimension	5.23±0.99	5.47±2.98	0.08

### 2.2 肺癌术后患者的提问信息总结

AI平台数据显示，在463例患者中，出院后7 d有119例（25.70%）患者主动对AI平台进行提问，282例（60.91%）仅提出1类问题，180例（38.88%）提出2类或以上问题。出院后14 d 344例（74.30%）患者无问题。患者问题可分为术后伤口类、咳嗽类、疼痛类、药物类、胸闷气短类、睡眠类、病理类以及其他等8类，各阶段（出院前、出院后7 d和出院后14 d）的具体提问频次见图2。

**图2:**
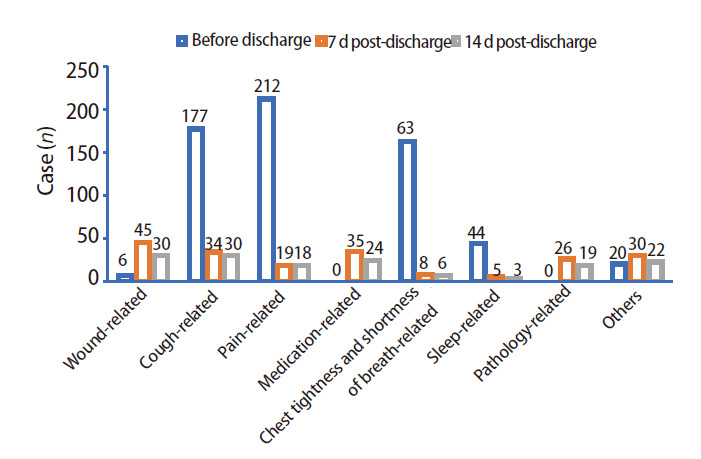
出院前、出院后7和14 d患者提问问题分析

### 2.3 肺癌术后患者咳嗽的影响因素分析

Leicester咳嗽问卷评分越高说明患者受到咳嗽的影响越小。结果显示，女性出院后7 d内咳嗽较男性多（P<0.01），且出院后14 d咳嗽明显减少，而男性无明显变化。老年患者出院后7 d内咳嗽与年轻患者相当，但出院后14 d内咳嗽较少（P=0.03）。吸烟患者在出院后7 d内（P<0.001）和出院后14 d内（P<0.01）咳嗽均少于无吸烟史患者。有家族史患者出院后7 d内咳嗽较少（P=0.04）。手术方式方面，出院后7 d内肺楔形切除患者咳嗽较少（P=0.02），出院后14 d内肺段切除患者咳嗽最少（P=0.04）。相比较其他手术方式而言，肺叶切除患者在出院后7 d内和出院后14 d内均有较多咳嗽困扰（表3）。

**表3:** 肺癌术后患者咳嗽影响因素

Influence factor		7 d post-discharge				14 d post-discharge		
		n=275	Cough score	P		n=218	Cough score	P
Gender	Male	118	16.66±2.76	<0.01		86	16.67±3.23	0.10
	Female	157	15.13±3.36			132	15.93±3.22	
Age	≤65 yr	179	15.82±3.13	0.78		150	15.90±3.23	0.03
	>65 yr	96	15.71±3.34			68	16.92±3.16	
BMI	<18.5 kg/m^2^	8	17.07±1.73	0.39		6	18.90±1.12	0.06
	18.5-23.9 kg/m^2^	130	15.80±3.11			113	16.38±2.94	
	>23.9 kg/m^2^	137	15.60±3.35			99	15.87±3.56	
Length of stay	≤3 d	105	16.07±3.37	0.33		78	16.05±3.62	0.76
	4-6 d	154	15.69±2.97			130	16.28±3.08	
	≥7 d	16	14.87±4.09			10	16.78±1.95	
Smoking history	No	197	15.37±3.30	<0.01		159	15.81±3.26	<0.01
	Yes	78	16.83±2.69			59	17.33±2.93	
Family history of lung tumors	No	221	15.59±3.31	0.04		171	16.09±3.37	0.25
	Yes	54	16.60±2.60			47	16.70±2.68	
Complication	No	142	15.63±3.34	0.40		117	16.33±3.18	0.58
	Yes	133	15.95±3.06			101	16.09±3.31	
Surgical approach	Lobectomy	90	15.08±3.18	0.02		73	15.43±3.40	0.04
	Segmentectomy	30	15.51±3.22			23	16.71±2.43	
	Wedge resection	155	16.25±3.15			122	16.60±3.20	
Number of nodules removed	1	209	15.90±3.06	0.30		158	16.22±3.361	0.98
	≥2	66	15.43±3.61			60	16.23±2.90	
Pathological results	Benign	35	16.50±2.39	0.16		26	16.87±3.08	0.28
	Malignant	240	15.68±3.30			192	16.13±3.26	
Lobe involved	1	229	15.85±3.11	0.46		174	16.16±3.39	0.58
	≥2	46	15.46±3.66			44	16.46±2.58	
Surgical side	Left	111	16.13±2.90	0.34		89	16.51±3.20	0.26
	Right	162	15.55±3.38			128	15.99±3.25	
	Both	2	15.69±4.56			1	19.95±0.00	
Number of incisions	Single hole	51	16.21±3.09	0.49		35	16.76±2.75	0.07
	Two holes	220	15.71±3.23			180	16.18±3.31	
	Thoracotomy	4	14.49±3.05			3	12.42±0.57	

## 3 讨论

### 3.1 肺癌术后患者出院后咳嗽问题与解决方案

胸腔镜下肺部手术是早期肺癌的重要治疗方式，基于当前加速康复外科（enhanced recovery after surgery, ERAS）理念的医疗模式，其中位住院时间仅为3 d^[3⇓-5]^。这意味着患者脱离医院在家的自身恢复期将延长，患者出院后所遇到的问题值得被研究。本研究表明，患者出院后主要关注伤口、药物、咳嗽及疼痛等问题，与陈曦等^[6]^的研究结果相似。出院后7 d内，患者对病理结果尤为关心；随着时间推移，问题逐渐减少，但至出院后14 d部分患者仍存在咳嗽问题。因此肺癌患者出院后面对的最大困扰就是咳嗽问题。

本研究发现，咳嗽对患者的影响在出院后7 d内较为明显，且在出院后14 d时仍然持续，提示术后咳嗽对患者的生活质量存在长期影响。研究^[7]^结果表明，女性、接受肺叶切除术的患者在出院后7和14 d内咳嗽较为明显，而老年患者在出院后14 d内咳嗽较少，提示不同人群对术后恢复的适应能力存在差异。因此，利用AI专病管理平台对术后咳嗽的发生诱因进行个体化分析与评估，针对出院后14 d内的咳嗽问题，应在围手术期采取预防措施，如术后预防肺部感染，指导患者有效咳痰并掌握呼吸功能训练。出院后通过AI平台推送个体化训练课程，并进一步推广可穿戴设备的居家应用，以增强患者自我管理能力^[8]^。

### 3.2 肺癌术后患者咳嗽可能的影响因素分析

Leicester咳嗽问卷分析显示，性别、年龄、吸烟史、家族史和手术方式等因素在出院后14 d内对咳嗽的发生和持续存在均有显著影响。女性出院后7 d内咳嗽较男性多，可能与女性气道敏感性、激素水平和免疫反应有关。老年患者在出院后7 d内与年轻患者咳嗽情况相当，但在出院后14 d内咳嗽明显减少，可能因为老年患者在术后日常活动有所调整，能更严格遵医嘱相关。

吸烟者在出院后7和14 d内咳嗽均少于无吸烟史者，可能由于长期吸烟导致气道对刺激的耐受性增加，咳嗽反射较迟钝。此外，家族史患者在出院后7 d内咳嗽较少，可能与遗传因素及对术后护理的重视程度相关。手术方式对咳嗽的影响也较为明显，肺楔形切除患者在出院后7 d内咳嗽较少，肺段切除患者在出院后14 d内咳嗽最少，而肺叶切除术患者在出院后7和14 d内咳嗽困扰较多（P<0.05）。这些结果提示我们，影响肺癌术后咳嗽的主要因素包括性别、年龄以及手术方式。针对女性、青年、肺叶切除患者，应制定更个性化的护理方案，如加强术后健康教育、呼吸功能训练，并通过AI平台实现个性化管理，以减少术后咳嗽的发生^[9,10]^。

### 3.3 肺癌术后患者提问情况现状与解决方案

专病管家服务会在患者出院后7和14 d主动推送周期量表，并提醒患者及时填写。患者出院后7 d健康日记填写率较高，但随时间推移逐渐降低，可能因患者健康问题减少，也可能与患者多为中老年人，受教育程度较低有关。出院前虽然接受了专病管家使用方法的培训，但部分老年患者仍较难适应该平台的使用。

这表明，在患者出院前，应加大对专病管家平台的引导和推广，使患者能够主动利用平台获取个性化健康管理信息。此外，出院后14 d患者部分类别的问题再次增多，反映出院前教育过于关注近期问题而忽视了长期存在的问题。因此，AI专病管家平台可针对出院后14 d后出现的长期影响问题进行归纳总结，在出院后14 d时对所有患者进行统一推送可能长期存在的问题的相关解答^[11]^。

AI专病管家平台减少了患者与医护人员出院后沟通的成本，减轻了患者在恢复期的心理压力。本研究通过AI专病管理平台对肺癌术后患者进行出院后管理，提高了患者的生活质量和医疗效率，有效干预了术后疼痛、咳嗽等常见问题。本研究还发现，性别、年龄、吸烟史等因素对术后咳嗽存在显著影响，为个性化护理方案的制定和AI技术在医疗领域的应用提供了支持。然而，本研究存在一定局限性，如样本量较小，仅基于单中心数据，且随访时间较短，难以评估长期效果；部分患者因年龄或教育水平限制，在使用AI平台时依从性较低；目前平台功能仍主要集中在健康教育和提醒，缺乏深度个性化干预。

未来研究应扩大样本范围，开展多中心试验，并延长随访周期，以评估AI平台在长期术后管理中的效果。同时，优化AI功能，结合大数据分析和可穿戴设备，提高个性化健康管理能力，拓展平台在其他术后管理领域的适用性，并改进用户体验，以提升患者的使用依从性和满意度。
